# Changes in knee joint destruction patterns among patients with rheumatoid arthritis undergoing total knee arthroplasty in recent decades

**DOI:** 10.1007/s10067-023-06620-w

**Published:** 2023-05-24

**Authors:** Ryutaro Takeda, Takumi Matsumoto, Yasunori Omata, Hiroshi Inui, Shuji Taketomi, Yuichi Nagase, Takuji Nishikawa, Hiroyuki Oka, Sakae Tanaka

**Affiliations:** 1 Department of Orthopaedic Surgery, Faculty of Medicine, The University of Tokyo, 7-3-1 Hongo, Bunkyo-ku, Tokyo, 113-8655 Japan; 2 Department of Rheumatic Surgery, Tokyo Metropolitan Tama Medical Center, 2-8-29 Musashidai, Fuchu-city, Tokyo, 183-8524 Japan; 3 Department of Rheumatology, Tokyo Metropolitan Bokutoh Hospital, 4-23-15 Koutoubashi, Sumida-ku, Tokyo, Japan; 4 Department of Clinical Motor System Medicine, 22nd Century Medical and Research Center, Faculty of Medicine, The University of Tokyo, 7-3-1 Hongo, Bunkyo-Ku, Tokyo, 113-8655 Japan

**Keywords:** Cluster analysis, Joint destruction, Rheumatoid arthritis, Secondary osteoarthritis, Total knee arthroplasty

## Abstract

**Objectives:**

This study aimed to investigate the trend of joint destruction patterns on knee radiographs of patients with rheumatoid arthritis (RA) undergoing total knee arthroplasty (TKA) over the past 16 years.

**Method:**

Medial joint space, lateral joint space, medial spur area, lateral spur area (L-spur), and femoro-tibial angle were obtained from 831 preoperative knee radiographs of patients with RA who underwent TKA between 2006 and 2021 using software capable of automatic measurements. Non-hierarchical clustering was performed based on these five parameters. Trends in the five individual radiographic parameters and the ratio of each cluster were investigated during the target period. Moreover, clinical data from 244 cases were compared among clusters to identify factors associated with this trend.

**Results:**

All parameters, except for L-spur, showed significant increasing trends from 2006 to 2021. The radiographs were clustered into groups according to the characteristic pattern of radiographic findings: cluster 1 (conventional RA type), with bicompartmental joint space narrowing (JSN), less spur formation, and valgus alignment; cluster 2 (osteoarthritis type), with medial JSN, medial osteophytes, and varus alignment; and cluster 3 (less destructive type), with mild bicompartmental JSN, less spur formation, and valgus alignment. The ratio of cluster 1 showed a significantly decreasing trend contrary to the significantly increasing trend in clusters 2 and 3. The DAS28-CRP of cluster 3 was higher than those of clusters 1 and 2.

**Conclusions:**

Radiographs of TKA recipients with RA are increasingly presenting osteoarthritic features in recent decades.

**Table:** 

**Key Points** *• Using automated measurement software, morphological parameters were measured from radiographs of 831 patients with rheumatoid arthritis who had undergone TKA in the past 16 years.* *• Cluster analysis based on the radiographic parameters revealed that the radiographs of patients with end-stage knee arthritis requiring total knee arthroplasty were classified into three groups.* *• In patients with rheumatoid arthritis who have undergone total knee arthroplasty in the past 16 years, the proportion of clusters with features of osteoarthritis and difficult-to-treat rheumatoid arthritis has increased, while the proportion of conventional rheumatoid arthritis has decreased.*

## Introduction

Rheumatoid arthritis (RA) is a chronic inflammatory disease characterized by persistent synovitis in multiple joints that subsequently causes joint destruction. Controlling inflammation using pharmacological therapy is the mainstay of treatment to prevent joint destruction in affected patients [1]. Over the past two decades, significant advancements in pharmacological therapy have emerged, such as the development of biological or targeted synthetic disease-modifying anti-rheumatic drugs (bDMARDs or tsDMARDs) and treat-to-target strategy aimed at tightly controlling disease activity [2]. These breakthroughs have dramatically improved the disease activity control of patients with RA. In Japan, infliximab, the first bDMARD, was approved for the treatment of RA in 2003. As of 2022, eight bDMARDs, including five tumor necrosis factor-alpha (TNF-α) inhibitors (infliximab, etanercept, adalimumab, golimumab, and certolizumab pegol), three agents with other mechanisms of action (tocilizumab and sarilumab [anti-interleukin-6 receptor antibody] and abatacept [cytotoxic T lymphocyte-associated antigen 4-Ig]), and five tsDMARDs (tofacitinib, baricitinib, upadacitinib, peficitinib, and filgotinib) are available for treating RA in Japan. A recent cohort study in Japan reported that more than half of patients with RA achieved remission [3]. Along with the expanded use of effective drugs, several database studies have indicated changes in RA-related surgeries, including a decreased incidence, changes in surgical demand, and changes in patient background factors [4–8].

Total knee arthroplasty (TKA) is the most frequent joint replacement procedure performed in patients with RA and has had accumulated evidence for the improvement of pain, quality of life, and disease activity [9, 10]. Because knee joint destruction directly affects patients’ ambulatory abilities and leads to great losses in activities of daily living and quality of life, TKA is highly valuable for patients with RA and knee involvement [11].

With advancements in pharmacological therapy, the population and characteristics of patients with RA who require TKA are expected to have changed. A retrospective study from a single institution in Japan revealed that the age of patients with RA who underwent joint replacement surgery increased and the preoperative serum C-reactive protein (CRP) levels decreased between 2004 and 2017 [12]. Some nationwide database studies demonstrated a decreased use of TKA procedures for patients with RA [4, 6, 7] in contrast to the worldwide increasing trend in the general population due to stable TKA outcomes and population aging [13–15]. Moreover, recent studies implicated an increasing trend of osteoarthritis (OA) features in the knee joints of patients with RA. A study in which artificial intelligence (AI) was used to classify the knee radiographs of patients with RA into RA or OA demonstrated that the latter increased between 2006 and 2020 [16]. However, that study did not specify the radiographic features that contributed to the classification by AI.

Another study comparing knee radiographs of patients with RA undergoing TKA between 1997 and 2003 and between 2013 and 2017 revealed that recent radiographs featured significantly higher rates of osteophytes and medially dominant joint space narrowing (JSN) as well as lower rates of bone erosion and geodes, suggesting that patients with RA tended to have OA-like features on radiographs after the pharmaceutical approval of bDMARDs in Japan [17]. Although this study revealed differences in individual radiographic features between the two time periods, it did not include comprehensive assessments of OA-like features.

Performing a cluster analysis based on measurable parameters on radiographs is one way to appropriately group joint destruction patterns. Studies have successfully established comprehensive patterns of joint destruction in the hands and feet of patients with RA using this method [18, 19]. However, to the best of our knowledge, no study has classified the joint destruction pattern of knees with RA using cluster analysis.

The current study aimed to clarify the changes in joint destruction patterns of knees of TKA recipients with RA in the recent 16 years. Here we performed a cluster analysis based on quantitative radiographic parameters obtained using an automated measurement software in the knees of TKA recipients.

## Methods

### Patients

**Fig. 1:**
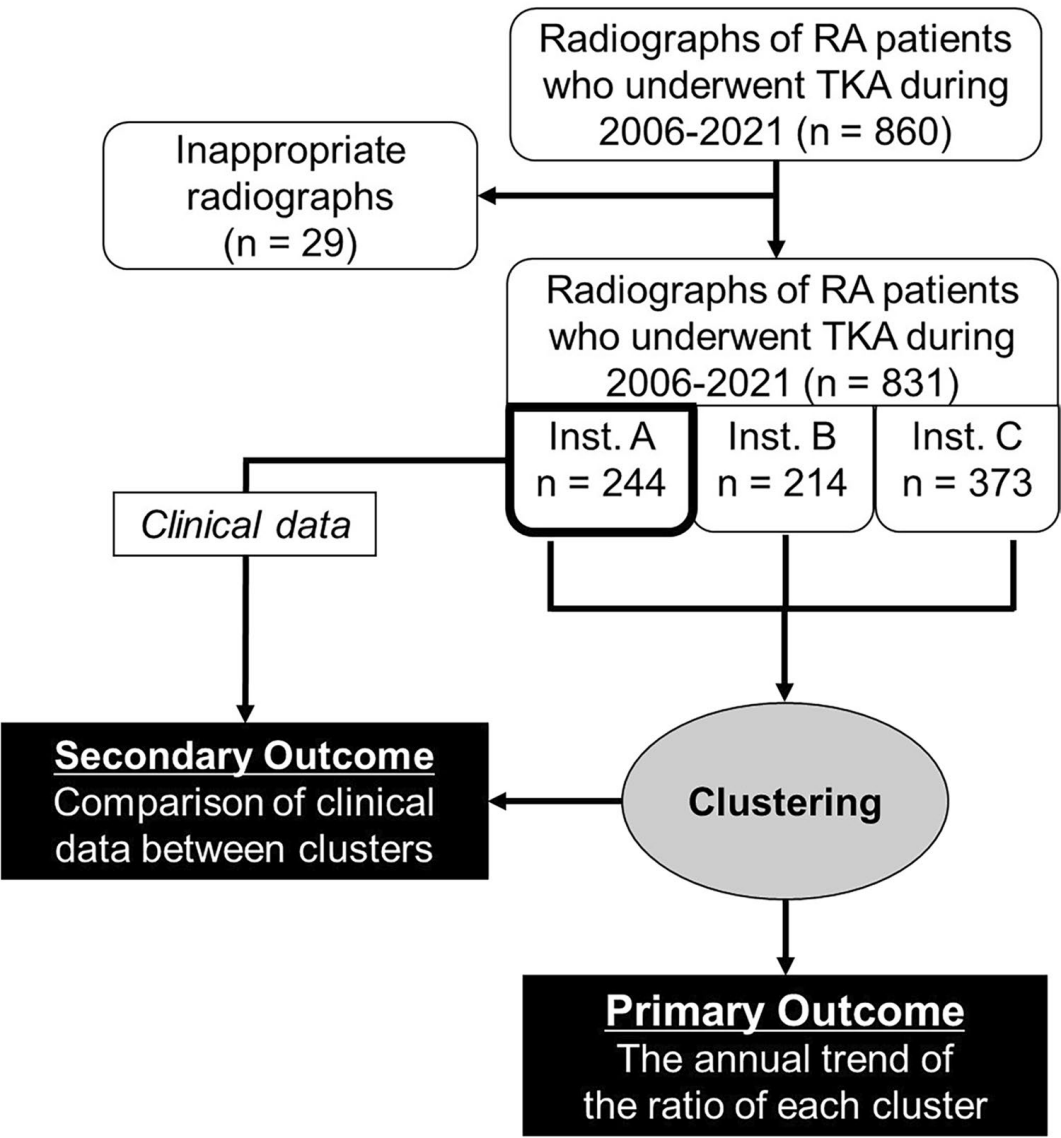
Flowchart of the study and patient inclusion process. A, B, and C represent the three participating institutions. RA, rheumatoid arthritis; TKA, total knee arthroplasty

A flowchart of the study is presented in Fig. 1. A total of 860 standing anteroposterior preoperative knee radiographs (675 patients) were included in accordance with the inclusion criteria below. In 57 cases, the knee radiographs included both knees; such radiographs were automatically separated along the midline and those of the preoperative side were used for the analysis. Among the 860 radiographs, 29 were excluded in accordance with the exclusion criteria below. Finally, 831 radiographs (646 patients) were included in the analysis.

#### Inclusion criteria

Surgical databases of three institutions were retrospectively reviewed, and patients with RA who underwent TKA between 2006 and 2021 and who did not have concurrent osteonecrosis of the femoral condyle were enrolled. Patients who underwent bilateral TKA were considered two separate cases, because the surgeries for the left and right knees were performed at different times, and the radiographs and clinical data were obtained just prior to surgery individually for the left and right.

#### Exclusion criteria

Radiographs which were inappropriate for automatic measurements by the software were excluded. The reasons considered inappropriate are as follows: partially containing the contralateral knee, containing implants, and indivisible using the automatic separation method.

#### Radiographs of normal knee and knee with primary OA

To obtain reference radiographic data for normal knees and knees with primary OA, radiographs were obtained from 20 patients who underwent second-look arthroscopy after anterior cruciate ligament reconstruction as a normal control and from 20 randomly selected patients with varus-type primary OA who underwent TKA at the University of Tokyo Hospital in 2020.

### Automatic measurement of radiographic parameters by KOACAD software

**Fig. 2:**
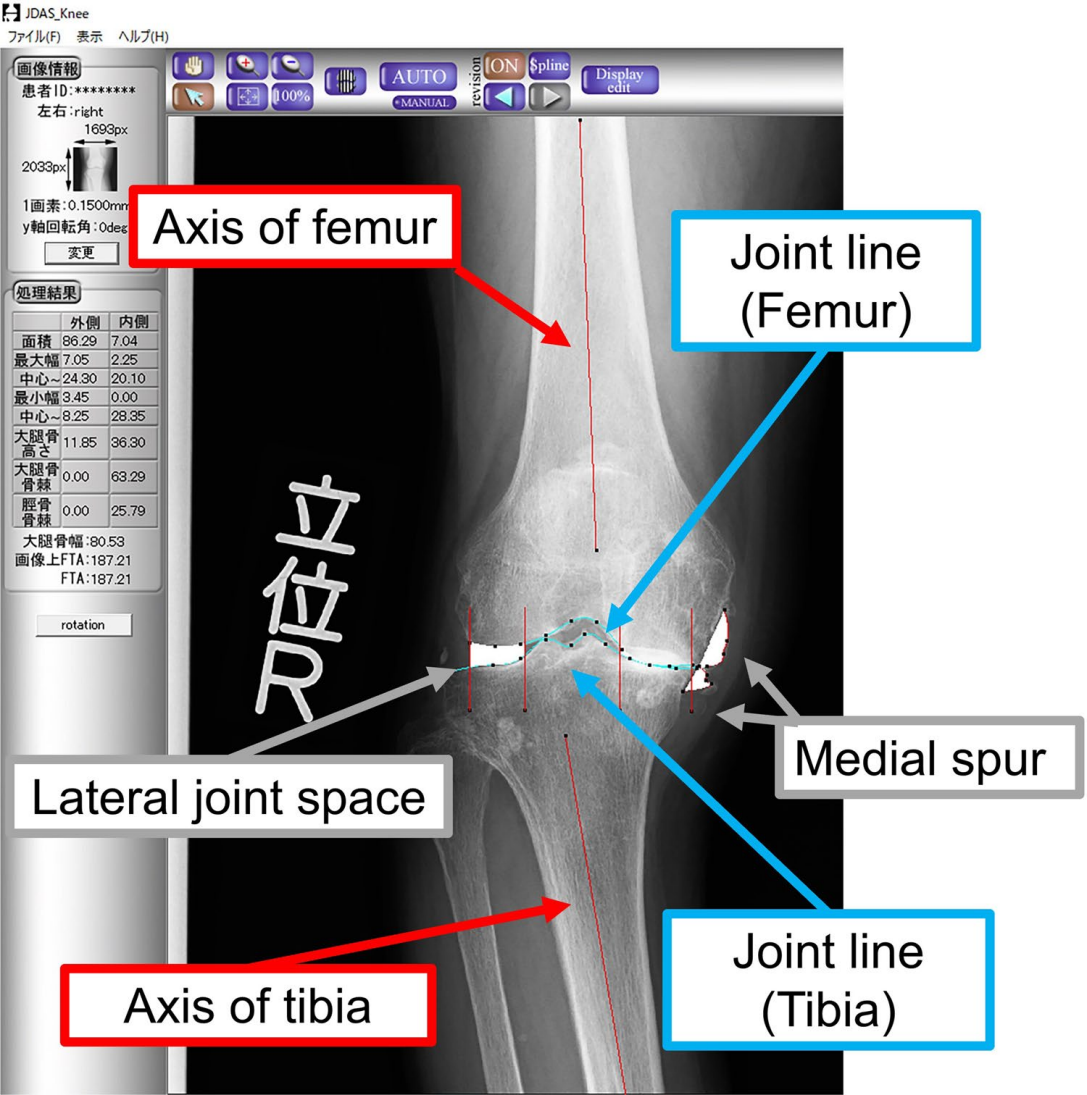
Automatic measurements of the radiographic parameters performed by knee osteoarthritis computer-aided diagnosis (KOACAD) software

Radiographic parameters of the medial joint space (MJS) [mm^2^], lateral joint space (LJS) [mm^2^], medial spur area (M-spur) [mm^2^], lateral spur area (L-spur) [mm^2^], and femoro-tibial angle (FTA) [°] were measured using knee OA computer-aided diagnosis (KOACAD) software (Inotech Co., Ltd, Hiroshima, Japan), which automatically measures these major parameters of knee OA on standing anteroposterior knee radiographs. KOACAD automatically extracts the tibial and femoral contours and estimates the medial and lateral edges of the femoral condyle and tibial plateau using a combination of various filters on the image. This allows KOACAD to evaluate the medial and lateral joint space as an area bounded by the joint line and joint edge. M-spur and L-spur are defined as the outer area of the joint edge, bounded by the femoral and tibial outlines. KOACAD also estimates the bone axis of the femur and tibia, allowing the calculation of FTA. KOACAD software has been validated for clinical use and approved as a medical device in Japan (Fig. 2) [20, 21]. When the reference points were inappropriate, each was manually modified by one of the authors (R.T.), a board-certified orthopedic surgeon.

### Analytical settings

The data processing and statistical analysis described in the following sections were performed using MATLAB 2022a (MathWorks, Natick, MA, USA). The level of statistical significance was set at *p* < 0.05. The normality of all continuous data was assessed using the Kolmogorov–Smirnov test.

### Trend analysis of age, sex, and radiographic parameters

The annual trends of age, MJS, LJS, M-spur, L-spur, and FTA in all 831 cases were tested using the Jonckheere–Terpstra test, a non-parametric rank-based trend test that reveals whether data show an increasing or decreasing trend over time. The annual trend in female ratio was tested using the Cochran–Armitage trend test, a trend test for ratios.

### Cluster analysis and annual trend analysis of cluster ratios

Non-hierarchical clustering was performed with the k-medoids algorithm of the 831 datasets containing five parameters (MJS, LJS, M-spur, L-spur, and FTA) [22]. The distance calculation was set at a standardized Euclidean distance. The optimized number of clusters was determined by comparing the Calinski–Harabasz index calculated based on the squared Euclidean distance, which indicates quality of clustering. Among the integer numbers from 2 to 6, the number that gave the largest Calinski–Harabasz index was considered the optimal number of clusters [23].

The annual trend in the number of patients in each cluster was tested using a linear regression model and the annual trend of the ratio of each cluster was assessed using the Cochran–Armitage trend test.

### Radiographic and clinical characteristics of each cluster

To clarify the radiographic characteristics of each cluster, the five radiographic parameters were compared among them using one-way analysis of variance (ANOVA), followed by multiple-comparison analysis using the Bonferroni method. To evaluate the clinical factors associated with the joint destruction patterns, we collected the clinical data from the medical charts of the 244 patients who underwent TKA at the University of Tokyo Hospital, including age, sex, body mass index (BMI) [kg/m^2^], duration of disease, dosage of prednisolone (PSL) [mg/day], dosage of methotrexate (MTX) [mg/week], use of bDMARDs for more than 6 months, C-reactive protein level (CRP) [mg/dL], presence of anti-cyclic citrullinated peptide antibody (ACPA), and Disease Activity Score-28 for RA with CRP (DAS28-CRP). The clinical data were compared among clusters. One-way ANOVA and Kruskal–Wallis test were performed for normally distributed continuous variables and non-normally distributed variables, respectively, while the chi-squared test was used to examine categorical variables. If a one-way ANOVA or Kruskal–Wallis test showed a significant difference between three clusters, the Bonferroni method was used to perform the multi-comparison analysis.

### Ethical considerations

The current multicenter retrospective study was approved by the research ethics committees of the University of Tokyo Hospital (no. 2674–4), Tokyo Metropolitan Tama Medical Center (no. 30–52), and Tokyo Metropolitan Bokutoh Hospital (no. 02–122).

## Results

**Fig. 3:**
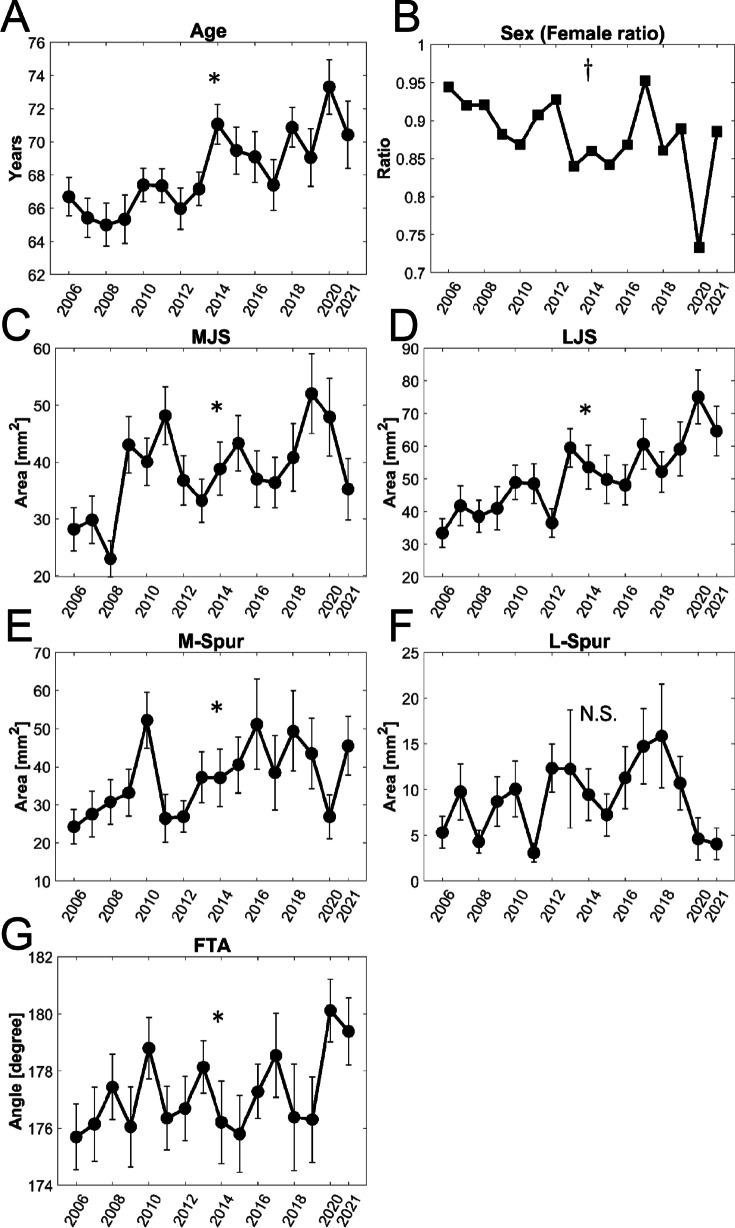
The annual trends in age, sex, and five radiographic parameters obtained using automatic measurement software from all 831 cases. The mean annual parameter values with standard errors are plotted. **A** Age, **B** sex (female ratio), **C** medial joint space (MJS), **D** lateral joint space (LJS), **E** medial spur area (M-spur), **F** lateral spur area (L-spur), **G** femorotibial angle (FTA). *p < 0.05, Jonckheere–Terpstra trend test. ^†^*p* < 0.05, Cochran–Armitage trend test. N.S., not significant

The annual trends in age, sex, MJS, LJS, M-spur, L-spur, and FTA obtained from the 831 cases are shown in Fig. 3. Significant increasing trends were observed in age, MJS, LJS, M-spur, and FTA from 2006 to 2021 (*p* < 0.0001, *p* = 0.0001, *p* < 0.0001, *p* = 0.0012, and *p* = 0.029, respectively). Meanwhile, the female ratio showed a decreasing trend (*p* = 0.035), while L-spur showed no trend during the study period (*p* = 0.081).

The Calinski–Harabasz indices were 337.0, 379.5, 327.7, 315.7, and 295.5 when the cluster numbers were set between 2 and 6, respectively. Because the maximum index was obtained with three clusters, clustering into three groups was performed using the k-medoids algorithm. The radiographs were classified into cluster 1 (397 radiographs), cluster 2 (222 radiographs), and cluster 3 (212 radiographs).

**Fig. 4:**
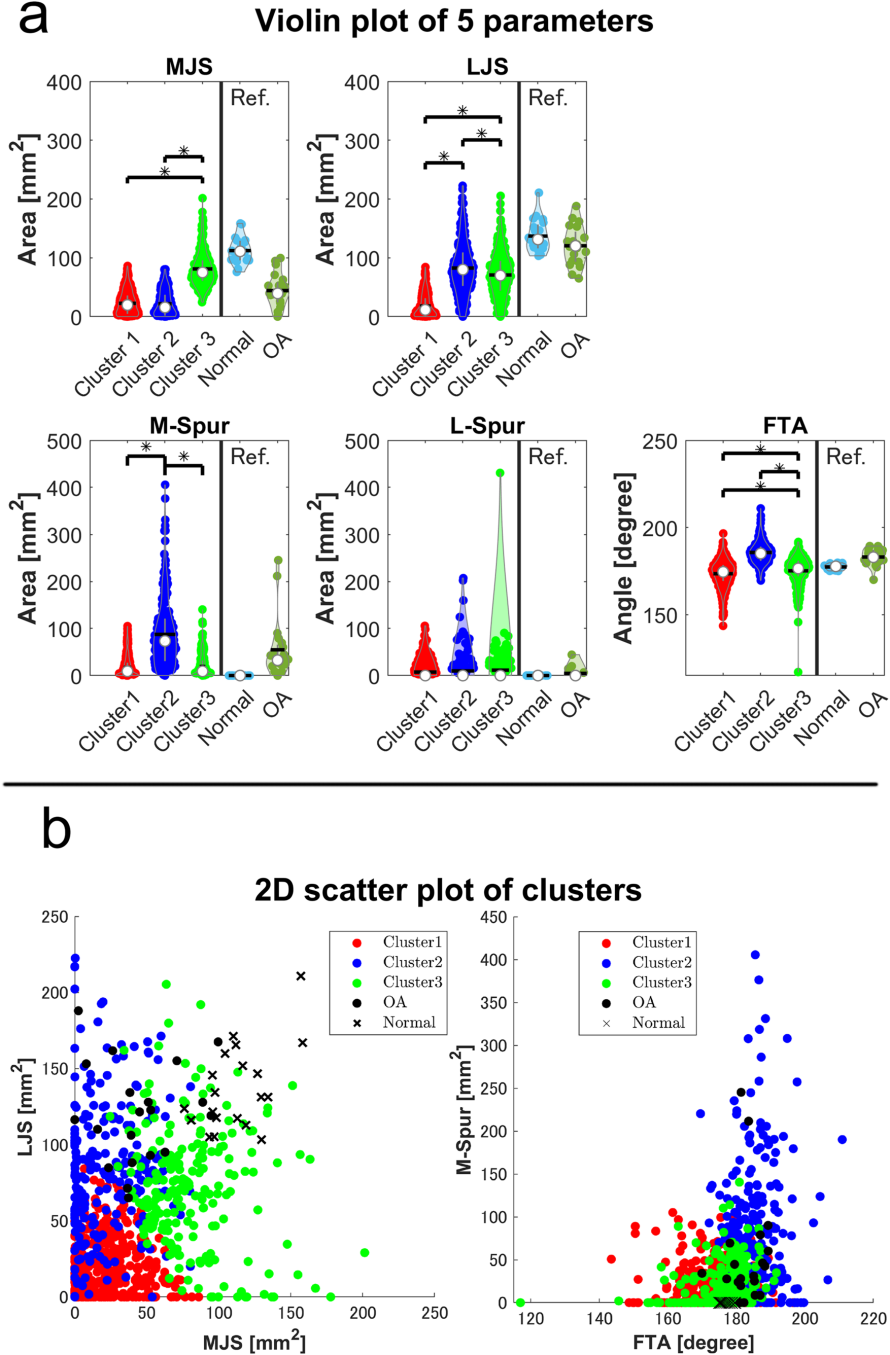
**a** Comparison of radiographic parameters among the three clusters. White circles and black horizontal bars in violin plots represent medians and means, respectively. The data of the 20 patients with primary OA and 20 normal controls are shown as reference. **b** 2D plots of clusters to visualize the joint destruction pattern. *p < 0.05, one-way analysis of variance with the Bonferroni multi-comparison method. FTA, femorotibial angle; LJS, lateral joint space; L-Spur, lateral bone spur; MJS, medial joint space; M-Spur, medial bone spur; OA, osteoarthritis; RA, rheumatoid arthritis; Ref., Reference

The five parameters used for clustering (MJS, LJS, M-spur, L-spur, and FTA) were normally distributed. The ANOVA results for MJS, LJS, M-spur, and FTA differed significantly among clusters (*p* < 0.0001, *p* < 0.0001, *p* < 0.0001, and *p* < 0.0001, respectively), whereas those for L-spur did not (*p* = 0.14) (Fig. 4a). To visualize the joint destruction pattern, 2D scatter plots with MJS and LJS as axes and 2D scatter plots with M-spur and FTA as axes were also shown (Fig. 4b). Each cluster was named according to their characteristic radiographic features revealed by this diagram as follows: cluster 1 (conventional RA type), valgus alignment (FTA: 173.4 ± 0.2°), bicompartmental JSN, and a low amount of bone spur formation; cluster 2 (OA type), varus alignment (FTA: 185.6 ± 0.4°), JSN of the medial compartment, and a high amount of bone spur formation on the medial side similar to the OA pattern shown as reference; and cluster 3 (less destructive type), valgus alignment (FTA: 175.1 ± 0.5°), and a low amount of bone spur formation with less bicompartmental JSN than cluster 1.

The clinical data of the three groups are described in Table 1. The data collection rates were 100% for all clinical data items except ACPA (62.7% [153/244]) and DAS28-CRP (73.7% [180/244]). Among the items analyzed, age and DAS28-CRP differed significantly among the three clusters (*p* = 0.0005 and *p* = 0.02, respectively). In the multi-comparison analysis, cluster 1 was the youngest and had an intermediate DAS28-CRP level; cluster 2 was the oldest and had the lowest DAS28-CRP level; and cluster 3 had an intermediate age and the highest DAS28-CRP level. There were no differences between clusters for sex, BMI, disease duration, PSL, MTX, biologic use, CRP, and ACPA (*p* = 0.26, 0.34, 0.78, 0.54, 0.68, 0.94, 0.15, and 0.88, respectively).

**Table 1:** Clinical data of the three clusters among 244 knees (196 patients) of one participating institution

	Cluster 1 (*n* = 92)	Cluster 2 (*n* = 81)	Cluster 3 (*n* = 71)	*p* value
Age, years	63.9 ± 0.9^a^	69.3 ± 1.0^a^	67.3 ± 1.0	0.0005
Sex, female/male (% female)	80/12 (87.0%)	74/7 (91.4%)	67/4 (94.3%)	0.26
BMI, kg/m^2^	22.7 ± 0.4	23.5 ± 0.4	22.7 ± 0.5	0.34
Disease duration, years	19.4 ± 1.3	18.7 ± 1.4	18.1 ± 1.5	0.78
PSL, mg/day	3.7 ± 0.3	3.3 ± 0.3	3.9 ± 0.4	0.54
MTX, mg/week	4.0 ± 0.4	3.5 ± 0.5	4.0 ± 0.5	0.68
Use of biologics, yes/no (% yes)	22/70 (22.7%)	21/60 (25.9%)	17/54 (23.9%)	0.94
DAS28-CRP	2.9 ± 0.1	2.7 ± 0.1^b^	3.2 ± 0.1^b^	0.02
CRP, mg/dL	1.2 ± 0.2	1.3 ± 0.2	1.7 ± 0.2	0.15
ACPA, positive/negative (% positive)	50/11 (81.9%)	37/8 (82.2%)	37/10 (78.7%)	0.88

Continuous variables are shown as mean ± standard error

^a^ Significant difference between clusters 1 and 2. ^b^ Significant difference between clusters 2 and 3

*ACPA*, anti-cyclic citrullinated peptide antibody; *BMI*, body mass index; *CRP*, C-reactive protein; *DAS28*; disease activity score; *MTX* methotrexate, *PSL*, prednisolone

**Fig. 5:**
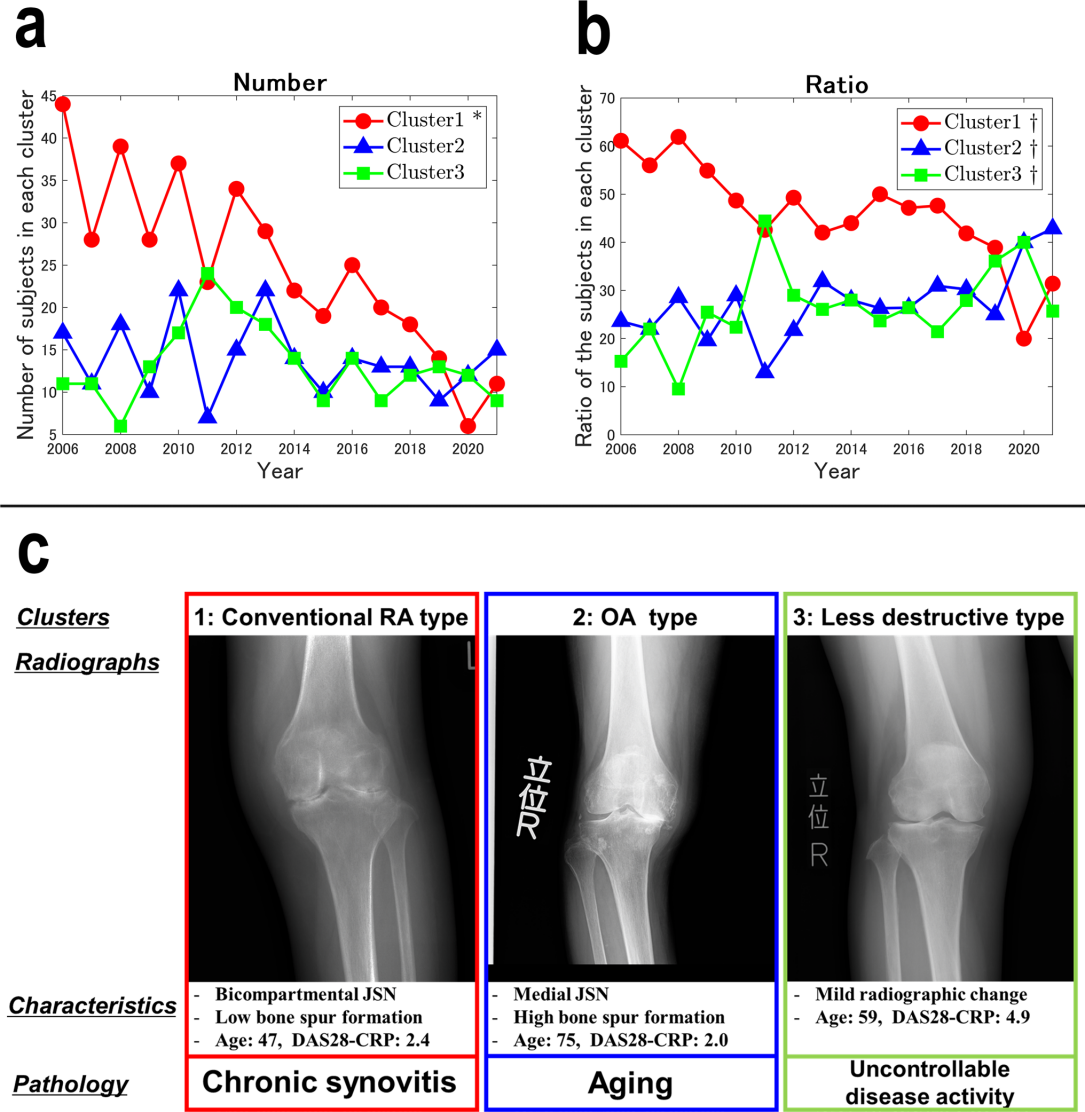
**a** The annual number of patients who underwent total knee arthroplasty at the three participating institutions by cluster. Cluster 1 decreased significantly from 2006 to 2021, while clusters 2 and 3 showed no significant trends. *p < 0.05, liner regression analysis. **b** The annual ratio of the patients by cluster. The ratio of cluster 1 showed a significant decreasing trend, while those of clusters 2 and 3 showed an increasing trend from 2006 to 2021. ^†^*p* < 0.05, Cochran–Armitage trend test. **c** Representative radiographs of three clusters and summary of their characteristics and estimated pathologies. OA, osteoarthritis; JSN, joint space narrowing; DAS28-CRP, disease activity score 28 with C-reactive protein

The number of patients in each of the three clusters from 2006 to 2021 is shown in Fig. 5a. The linear regression analysis revealed a significant decrease in the number of patients classified in cluster 1 (*R* = 0.78, *p* < 0.0001), while the numbers of patients classified into clusters 2 and 3 showed no significant trend (cluster 2: *R* = 0.052, *p* = 0.39; cluster 3: *R* = 0.019, *p* = 0.61). The ratio of the number of patients in each cluster to the total number of patients in each year is shown in Fig. 5b. The ratio of cluster 1 showed a significant decreasing trend (*p* < 0.0001), whereas those of clusters 2 and 3 showed significant increasing trends (*p* = 0.015 and *p* = 0.0064, respectively).

## Discussion

The current study revealed the annual trend of OA-related radiographic parameters evaluated quantitatively in knees with RA over the past 16 years. The cluster analysis based on these radiographic parameters divided the RA knee radiographs of TKA recipients into three clusters with different radiographic and demographic features: cluster 1, with the radiographic features of conventional RA; cluster 2, with the radiographic features of OA and low RA disease activity; and cluster 3, with mild radiographic changes and high RA disease activity (Fig. 5c). In contrast to the increasing trends noted in clusters 2 and 3, the ratio of cluster 1 demonstrated a decreasing trend from 2006 to 2021.

In the current study, the amount of medial spur showed a significantly increasing trend during the study period. Osteophyte formation has been considered incompatible with RA, which is characterized by excessive bone resorption by osteoclasts over bone formation by osteoblasts. Molecular biological studies have reported that inflammatory cytokines, such as interleukin-6 or TNF-α, hamper osteophyte formation through Dikkopf-1 protein expression [24]. In clinical research, low CRP levels (< 0.3 [mg/dL]) were reportedly associated with osteophyte formation in the knees of patients with RA before TKA [25]. The overall improvement in disease activity due to recent advancements in pharmacotherapy might contribute to the increasing trend of medial bone spurs.

Uniform JSN of the medial and lateral compartments is considered a characteristic of knees with RA in contrast to the medial-dominant JSN of knees with OA [26]. In the current study, the incidence of LJS increased by approximately twofold over 16 years, suggesting that the proportion of knees presenting the conventional RA pattern is decreasing as that of knees presenting the OA pattern is increasing. This consideration was also supported by the absolute and relative decrease in cluster 1 (conventional RA type) and the relative increase in cluster 2 (OA type). The increase in LJS noted in the current study is consistent with the findings of a previous study comparing the characteristics of knee radiographs of patients with RA who underwent TKA between 1999 and 2002 and between 2013 and 2017, which reported a decreased ratio of bicompartmental JSN from 68.7 to 47.9% and an increased ratio of medial-dominant JSN from 20.9 to 45.3% over time [17]. The current study also revealed an increasing trend of MJS; however, the gradient was not as steep as that of LJS. We believe that this increasing trend of MJS might reflect the absolute decrease of cluster 1 with severe JSN and the relative increase of cluster 3 with mild radiographic changes during the study period.

An increasing trend of FTA was also revealed in the current study, reflecting the decrease in cluster 1, of which the mean FTA was 173.4°, and the relative increase of cluster 2, of which the mean FTA was 185.6°. Although no study has investigated the general characteristic of knee alignment in RA, there are several previous studies reporting FTA on the radiographs of patients with RA before TKA in the pre-biological era. A previous study revealing the correlation between FTA and osteoporosis in 99 radiographs of knees with RA before TKA between 1990 and 2003 reported that 78% of patients had normal or valgus alignment and that the mean FTA was 176.2° [27]. Another study investigating 95 knees in TKA recipients with between 1979 and 1981 reported that 69% of cases had valgus alignment and 25% had neutral alignment [28]. A previous study including 71 cases of TKA in patients with RA between 1979 and 1982 reported that the median of preoperative FTA was 6° valgus [29]. These previous studies suggested that the typical cases of conventional RA before the biologic era were normal or valgus alignment.

In addition to demonstrating each OA-related radiographic parameter, the cluster analysis in the current study demonstrated the relative increase in the radiographic pattern with comprehensive OA-like features and the absolute and relative decrease in the pattern with conventional RA-like features. The existence of knees with RA with mild radiographic changes (cluster 3) was cleared up separately from those of cluster 1 (conventional RA type) and cluster 2 (OA type). Cluster 3 showed a relatively increasing trend during the study period and demonstrated the highest DAS28-CRP among the three groups. Considering the clinical and radiographic characteristics, the patients in cluster 3 underwent TKA despite mild joint destruction on radiographs due to the symptoms associated with inadequately controlled disease activity. Despite the recent advancements of pharmacological therapy, a significant proportion of patients with RA are symptomatic with difficult-to-treat RA (D2TRA). A previous retrospective cohort study in Japan revealed that the prevalence of D2TRA was 7.9% between 2011 and 2020 [30]. The demand for TKA in patients with RA will still decrease due to pharmacological therapy advancements and will not disappear because of the constant existence of OA knees in patients with RA and D2TRA.

Changes in patient demographics, including age at surgery and sex, were observed in the current study (Fig. 3). The increasing trend of age at TKA observed in the current study was consistent with the report by Asai et al., which revealed an increasing trend of age at the time of surgery in patients with RA between 2004 and 2018 at two institutions in Japan [31]. The increasing trend in age can be explained by the decrease in cluster 1 (with the youngest average age) and the relative increase in cluster 2 (with the oldest average age). The trend in female ratio in the current study decreased from approximately 95 to 80–85% in this decade and a half, possibly due to the increasing age at the time of surgery. The female/male ratio of patients with RA in Japan is 75% overall, and a tendency to decrease with age was observed from a maximum of 81% among patients 50–59 years of age to a minimum of 66% after 80 years of age [32].

Another possible reason for the increase of female ratios is an increasing number of patients with RA undergoing TKA who had pathogenesis of knee OA. The ROAD study (the Research on Osteoarthritis/Osteoporosis Against Disability study), a population-based cohort study performed in several communities in Japan, reported that the female ratio of patients with symptomatic knee OA with radiographs graded 3 or 4 in the Kellgren–Lawrence grading system was 79.0%, comparable to the female ratio of patients with RA who underwent TKA in recent years presented in the current study [33]. Although no difference in sex distributions among clusters was demonstrated in the current study, the decrease in cluster 1 and the relative increase in cluster 2 might have contributed to the convergence of the female ratio in patients with RA to the level of that in patients with OA.

The current study has several limitations. First, it lacked parameters representing joint erosion because KOACAD software evaluates OA-related features and cannot measure erosion degree. However, the quantitative evaluation of erosion is impractical, especially in destroyed joints requiring TKA. Second, the collection of clinical data, such as the presence of ACPA and DAS28-CRP, was limited by the retrospective medical chart review. In addition, the collection of clinical data was only from one of three institutions. Despite these shortcomings regarding clinical data collection, we consider their effect on the current study’s results limited because the data acquisition rates exceed 60% for ACPA and 70% for DAS28-CRP with no arbitrary selection of the data.

In conclusion, the trend analysis of radiographic findings in the knees with RA of TKA recipients over the last 16 years revealed a decrease in the typical radiographic features of RA. The cluster analysis demonstrated the absolute and relative decrease of knees presenting typical RA features, including a relative increase in those presenting secondary OA-like changes in older patients with well-controlled disease activity and a relative increase in those with mild radiographic changes but with uncontrolled disease activity in the era of biologics.

## Data Availability

The datasets except for private information are available at https://doi.org/10.17632/97cwmhfmtf.5. The radiographs used in the current study are available from the corresponding author on reasonable request.

## Code availability

The analysis code is available online at https://doi.org/10.17632/97cwmhfmtf.5.
